# Supplementary material for: The impact of the SARS-COV2 infection on the disorder of consciousness rehabilitation unit
Source: PLoS One. 2021 Jun 30;16(6):e0253958. doi: 10.1371/journal.pone.0253958 (PMC8244854; doi:10.1371/journal.pone.0253958)
Supplement: Attachment — Submitted filename: Response to reviewers.doc [file pone.0253958.s002.doc]

Messina, Apr 22, 2021

Dear Editor,

detailed responses to the reviewers’ questions appear below:

Reviewer #1:

We thank the reviewer for the kind and appreciative words. We revised the paper, as suggested.

- we included steroid regimen use;

- we included BMI;

- we included metabolic profile.

Reviewer #2:

We thank the reviewer for the time spent to revise the manuscript. We revised the paper, as suggested.

Statistical section, already reports what was requested by the reviewer:

- statistical method used: "*Mean and standard deviations (SD) were used for normally distributed data and median and range for data that were not normally distributed. Continuous variables were compared by using Wilcoxon rank sum test. Proportions for categorical variables were compared using χ^2^ test. Because of the small sample size, ordinal, interval and ratio measures are presented as median and range*";

- statistical software used: "*All statistical analyses were performed using R (version 3.3)".*

We thank you again for your consideration of our work and look forward to hearing from you in due course.

Sincerely,

Rosella Ciurleo PharmD, PhD

IRCCS Centro Neurolesi “Bonino-Pulejo”

S.S. 113, Via Palermo, C.da Casazza

98124 Messina, Italy

Phone: + 39-090-60128109

Fax: + 39-090-60128850

e-mail: rossella.ciurleo@irccsme.it
